# A Field Theory Perspective on Journalist–Source Relations: A Study of ‘New Entrants’ and ‘Authorised Knowers’ among Scottish Muslims

**DOI:** 10.1177/0038038517696220

**Published:** 2017-04-03

**Authors:** Michael B Munnik

**Affiliations:** Cardiff University, UK

**Keywords:** Bourdieu, field theory, journalism, journalist–source relations, media meta-capital, Muslims, representation, Scotland, sources, Twitter

## Abstract

In this article, I apply Bourdieu’s field theory to research on the trajectories, strategies and relations of sources and journalists. I argue that the relational emphasis of field theory, modified by the concept of media meta-capital, can be a fruitful way of examining the social context in which representations of Muslims are produced. This advances scholarship that relies too heavily on content analysis to support judgements about news representations of Muslims. I use examples from original fieldwork in Glasgow to discuss the capital, autonomy and heteronomy of Muslim sources who are ‘authorised knowers’ and ‘new entrants’ in their source communities. These various positions are evident in their relative success in managing journalist–source relations, which encompass ‘legacy’ media platforms and emerging communication tools such as Twitter. The field theory perspective exposes relations that contribute to the work of representation but are invisible to other forms of analysis.

## Introduction

There is now a significant sociological literature on the study of news media and journalistic content (e.g. Couldry, 2012; Galtung and Ruge, 1965; Schlesinger, 1987; Schudson, 2003). Too often, however, studies of this subject focus on the outputs and the ideological bent they betray rather than the processes by which these outputs are produced. In this article I seek to address this oversight through a theoretically informed intervention that traces how relationships between sources and journalists can determine the limits to which a source may influence the shape of a story or whether an event becomes a story at all. Scholars considering the social context of journalism have advocated for research that attends to these relationships (Gans, 1980; Schlesinger, 1990). Pierre Bourdieu’s field theory places relationships at the fore of its enquiry and would seem therefore a good tool for use in this exploration. The field approach separates journalists and sources into discrete spheres, and within these fields, scholars map positions, the act of taking positions and the relationships between positions. This approach has value for micro-studies of news organisations, but as Bourdieu (1998) suggested, what is interesting for a wider sociological audience is the impact the journalistic field has on other fields. Nick Couldry’s (2003) concept of ‘media meta-capital’ offers a theoretically consistent way to examine journalist–source relations in field perspective.

Applying these concepts to the study of Muslim sources in particular addresses a deficit in the scholarship of Muslims in British society. Studies often rely on content analysis to support judgements about the representation of Muslims in the UK, identifying news organisations as carriers of Islamophobia that, in some analyses, craft intentionally negative portrayals of Muslims (Commission on British Muslims and Islamophobia, 1997; Morey and Yaqin, 2011; Saeed, 2007). Periodic content studies have reached similar conclusions, albeit with increasing technological sophistication, larger samples and more rigorous and nuanced analysis (Baker et al., 2013; Bleich et al., 2015; Moore et al., 2008; Poole, 2002). However, the consistency of these findings suggests it is time to ask new questions of the subject (Munnik, 2015), and a key area to expand on is what Simon Cottle (2007) calls ‘the problem of inference’, asking how these representations come about. How can we know what motivates journalistic coverage of Muslims if we do not hear from journalists; how can we know whether relations between journalists and Muslims (imagined as separate, incompatible spheres) are fraught if we do not know what those relations look like? By qualitatively studying relations between journalists and Muslim sources, we are able to look past the *product* of the news, instead examining the *processes* by which it is created and the participation of various actors in its creation. In doing so, we will find that the actions of Muslim sources and their relationships with news workers help shape media representations of Muslims.

## The Journalistic Field

Bourdieu’s concept of fields is a general sociological model that is relevant to the work of thinking about journalism and specifically journalist–source relations. Here, I consider the identification of a field as a primarily methodological question (Hilgers and Mangez, 2014). Bourdieu wrote little about journalism: *On Television and Journalism* (1998) is a slender essay, written to be read aloud and concerned with French examples. Media specialists found its arguments unempirical, ungeneralisable and unremarkable (Benson and Neveu, 2005b; Marlière, 1998). More rigorous is Bourdieu’s posthumous essay (2005) in which he invoked journalistic, political and social science fields and discussed their relations. Bourdieu’s emphasis here was on the highly heteronomous character of the journalistic field: journalists are beholden to agents in other fields in order to carry out their work. This essay helps us to consider journalist–source relations, but first let us see how scholars have established and developed the idea of a journalistic field.

Given the paucity of direct analysis of journalism, Erik Neveu (2007) suggests that a more fruitful course of action for media scholars is to turn to Bourdieu’s writings on education and art, translating his concepts into the journalistic field. In this corpus, Bourdieu proposed the concept of field as a spatial metaphor for analysing objective relations within a bounded social setting. For him, ‘every position, even the dominant one, depends for its very existence, and for the determinations it imposes on its occupants, on the other positions constituting the field’ (1983: 312). These objective relations are ‘invisible, or visible only through their effects’ (1983: 311). Bourdieu designed this approach to evade a commitment to either internal reading, which focuses exclusively on the unit of analysis (e.g. a literary text) with no reference to its context; or external analysis, which reduces agency, submitting it to anonymous structures. Bourdieu chose instead ‘a social universe’ that puts the producers of works in context (2005: 33, cf. 1996: 193–206). The field is first and foremost, then, a concept for understanding social phenomena through relationships.

Consonant with his emphasis on positions, Bourdieu (1993: 189) encouraged analysis not of ‘ordinary biographies’ of people but trajectories of agents, or ‘the series of positions successively occupied by the same [agent] in the successive states of the [relevant] field’. We can evaluate this trajectory by assessing the agent’s stores of capital, which can alter over time in her struggles within the field, and her habitus, which both shapes and is shaped by the collective activity of the field and which directs the choices of the agent without being determining. The distribution of capital gives the field its structure, but capital is unevenly distributed, and those whom Bourdieu (1999: 37–38) termed ‘new entrants’ must make use of succession or subversion strategies to wrest influential positions from established actors who, in turn, employ conservation strategies to retain their positions.

Scholars have persuasively applied these tools to the study of journalism, including the ‘classical’ newsroom ethnography and macro-level political communication studies (Benson and Neveu, 2005a; Schultz, 2007; Willig, 2013; Willig et al., 2015). David Ryfe (2012) implicitly adopts them when studying attempts by US daily newspapers to forestall or embrace digital technologies: his categories of habits, investments and definitions mirror Bourdieu’s habitus, illusio and doxa (see Benson and Neveu, 2005b), and his work bridges empirical ethnographic study and wider structural analysis.

Not all media scholars are satisfied that fields are fit for purpose in the study of journalism. Roger Dickinson (2008) endorses Bourdieu’s theory as a means of connecting the thick descriptions of newsroom ethnographies to structures, but for studying journalistic practice, he prefers the less abstracted concept of social worlds devised by US sociologist Howard Becker (1982). Dickinson accepts Becker’s complaint about Bourdieu – that fields are bloodless, populated by agents rather than people and reliant on invisible objective relations rather than observable interactions (Becker and Pessin, 2006). For his part, Bourdieu (1996: 205, emphases in original) credited Becker with bringing wider context to the study of cultural production but nevertheless insisted that ‘the artistic field is not reducible to a *population*, that is to say, to the sum of individual agents linked by simple relations of *interaction*’. Wendy Bottero and Nick Crossley (2011: 102), in comparing the two models, find that interaction necessarily underpins the structural analysis on which Bourdieu insisted – that he ‘is tacitly relying upon an understanding of differential association for the intelligibility of his account’. Bourdieu’s sin, then, was not ignoring ‘people’ for the sake of ‘agents’, as Becker portrays it, but assuming their presence without crediting their agency in his theory.

## Field Theory and Journalist–Source Relations

In his posthumous essay, Bourdieu (2005) posited the idea of a journalistic field and immediately placed it in relation to other fields. I suggest that this encourages us to use Bourdieu’s concepts to scrutinise journalist–source relations, thereby exposing social processes at work behind the visible representations in published news texts. But media sociologist Nick Couldry (2003) has noted that the relation of the journalistic field to other fields seems to break Bourdieu’s own ‘rules’ for how this theory is applied. A ‘crucial difficulty’ for Couldry (2003: 661, emphases in original) is the question, ‘how exactly have representations made by actors in *one* field come to have such influence on the actions and thoughts of others in *another* field?’ Couldry (2003: 667–668) sees the influence of journalistic forms on the positions and position-takings of those in related fields (especially the political field) as an expression of a wider ‘media power’ or, as he proposes, ‘media meta-capital’ which constrains other fields. This constraint seems to contradict the heteronomy which Bourdieu identified in journalism: how can the media constrain other fields when it is reliant on the co-operation of those fields to conduct its own work? A rigorous study of these dynamics, however, would show an ambivalent relationship in which both trends operate, at different levels and different times.

The notion of ‘other fields’ is appropriate to bounded spheres of collective activity, such as politics or social science, but we may be stretching fields too far by invoking ‘sources’ as a generic field. Ida Willig and colleagues (2015: 3), in introducing a collection of recent articles relating field theory to new media, caution that field ‘should not be an omnibus concept but instead an open question for empirical analysis’. The literature on journalist–source relations provides such analysis, whether it flies the flag of Bourdieu or not. Richard Ericson and colleagues (1989), for example, include political, legal and law enforcement agents in their study of news sources and the control they can exert on coverage of crime in Canada. Similarly, David Miller and colleagues (1998) examine the activity of government bureaucracies, the nationally administered health service and voluntary sector groups concerning the reporting of AIDS in Britain. A strict Bourdieusien analysis ought to separate these fields, but these scholars sensibly recognise that the sources in question are bounded, in however ad hoc a manner, by their relationships to journalists in the formation of news content.

Some early newsroom ethnographies considered sources but instrumentally, isolating the focus on the journalists, their work and their needs. Jeremy Tunstall (1971) noted sources as one of three audiences which journalists consider and to which they are beholden when crafting stories. Mark Fishman (1980) observed how bureaucratic sources have abetted the formation of ‘beats’ or ‘rounds’ for journalists as a way of canalising thought by limiting choices for exploration and conferring upon themselves the status of ‘authorised knowers’, a term redolent of Bourdieu’s (1990) use of ‘legitimacy’. Herbert Gans (1980) made sources far more central to journalistic practice, calling them the most significant consideration for journalists when deciding what is news; he did not incorporate sources into his study but urged scholars to develop this seam of research (1980: 360, fn. 3).

Philip Schlesinger (1990) answered Gans’s call, critiquing previous studies as ‘media-centric’ and pointing to the significance of the strategies sources employ to gain news coverage. Inspired by the research of Ericson and colleagues (1989), Schlesinger conceives of news coverage as an accomplishment, dependent on the collective action of sources in competition with other sources for the attention of journalists who are themselves beholden to other journalists, their superiors and their audiences. Schlesinger applies Bourdieu’s theory to expose this work, naming the communities both of journalists and of sources as fields and marshalling capital as a way of accounting for the unequal but contingent access sources have to the definition of an issue *as* news and *in the* news.

Since Schlesinger’s intervention, more scholars have seen the value of connecting these parties and scrutinising their relationship in practice rather than merely in print (e.g. Davis, 2009; Deacon, 1996; Miller et al., 1998). Few have made explicit use of Bourdieu’s fields in their analysis, but the concept’s relational focus helps this line of inquiry; this is especially evident in a study by Patrick Champagne and Dominique Marchetti (2005) that examines AIDS reporting and the tainted blood scandal in France by examining the collective action of medical scientists, health correspondents and generalist reporters. Notwithstanding their caution about the slippery use of the term ‘field’, as noted above, Willig and colleagues (2015: 3) observe that:

[a] social space comes to work as a field when the institutions and characters that enter it are part of and feel its stakes, values, and debates, when one cannot succeed in it without a minimum level of practical or reflexive knowledge of its internal rules and logics.

Studies that examine journalist–source relations do precisely this work, and for this reason, I submit that Bourdieu’s concept of field and attendant tools such as capital, habitus and trajectory provide a vocabulary and framework that allow social researchers to examine and evaluate these complex relationships.

## An ‘Authorised Knower’ and a ‘New Entrant’

Between August 2012 and March 2014, I interviewed 30 participants in Glasgow, Scotland, 18 of whom I class as sources and 12 as journalists, though these categories are provisional and porous. The journalists worked for several news organisations, encompassing broadcast media, broadsheet newspapers and tabloid newspapers, each with digital expressions. Sources came from several professional fields, including politics, law and civil society; they either identified as Muslim or were engaged in social activity concerning Muslims. I observed two newsrooms and a Muslim women’s centre, and I enriched this fieldwork with the reflexive inclusion of my prior professional experience (Munnik, 2016) and documentary analysis, including relevant published news reports, internal documents, annual reports and communications through social media.

I chose not to restrict my sample of journalists to a single organisation, following the notion of field as a ‘meso-level’ collective (Dickinson, 2008). Similarly, my sample of news sources is eclectic, demonstrating that Muslim sources in Glasgow have increased both in number and in diversity in the last 15 years (Munnik, 2017). News sources, as we shall see, do not restrict themselves to one news organisation when communicating and promoting a story or comment; rather, they consider the range of media options and choose strategically whom to contact and how. For their part, journalists consider their counterparts at competing organisations when making decisions about coverage (Anderson, 2013; Tunstall, 1971), so I have treated Glasgow as a journalistic field, with both organisations and individual news workers occupying positions relative to each other within that field. Falling short of declaring a loosely formed ‘Muslim source field’, I nonetheless employ the tools of field analysis to the production of representations of Muslims in news texts, with sources who identify as Muslim taking positions as contributors to that process.

My sample was purposive, including participants for their relevance to the research question rather than their representative qualities. I targeted journalists and sources for inclusion, and I asked them for recommendations, which led me to others who were similarly involved in the production of news about Muslims in Glasgow. Studying the news media involves public material, and in many cases, the identities of people, groups and institutions are so entangled in the illustration that it would not be practical to anonymise them. To prepare for this, I provided every participant in my study with a consent form, which included an option for them to request that I disclose or protect their identity, with no penalty to their involvement in the study either way. Most consented to identification and are, when appropriate, named in this article. Twitter handles are included, as these communications are part of the public record and openly accessible. However, I make no necessary link between an individual or institutional account and the user behind the computer; this last step is unverifiable, so I attribute this activity to ‘the user of the account’. Most interviews were conducted in person, commonly at a coffee shop or the participant’s workplace or home, with the remainder conducted by phone or, in one instance, Skype. Two-thirds of the interviews were recorded and transcribed, and for the remainder I took notes. I later summarised each interview and sent it to the participant for corroboration and, in some circumstances, the opportunity to ask follow-up questions. Participants were not invited to query or alter my analysis of their responses. I present two cases from this research to illustrate how field theory helps us examine journalist–source relations. The first concerns an individual – Aamer Anwar, a source participant; the second is a set of interactions between journalists and sources on Twitter.

### The Authorised Knower: Capital and Trajectory

Aamer Anwar is a lawyer in Glasgow who has built a career defending human rights cases. His confident management of media relations is a product of his history, and a brief account of his trajectory gives us the context in which to analyse these relations. Anwar said he became politically active as a university student in the late 1980s, demanding equality policies such as blind marking after his flatmates – fellow Asian students – told him they were receiving lower marks because of their racialised identification. While flyposting in support of his cause, Anwar was beaten by two police officers; he sued the police service, alleging that the attack was racially motivated, and he won his case in 1995. After this, he began to work for the Commission for Racial Equality and studied law.

Social justice and equality have defined his work. He was active in the case of the murder of Surjit Singh Chhokar, a man from Lanarkshire who was killed in 1998. Anwar was among those who labelled the case ‘Scotland’s Stephen Lawrence’, a reference to the 1993 murder of a young black man in London; a judicial inquiry into Lawrence’s case was underway when Chhokar was murdered, making the reference timely and, for journalists, easily contextualised. As with the Lawrence case, the treatment of Chhokar’s murder was for Anwar evidence of institutional racism. Anwar has also led campaigns against the Scottish Defence League, an anti-Muslim and, arguably, racist group affiliated with the English Defence League.

Anwar identifies as Muslim, and although he said he dislikes the journalistic commonplace phrase ‘Muslim lawyer’ in news articles about him, he has defended clients whose religious identification was related to their arrest and prosecution, including a group of Algerian nationals charged with terrorism offences in Edinburgh in 2002 and Mohammed Atif Siddique, a young Scottish man from Clackmannanshire who was charged and convicted under terrorism laws and later acquitted on appeal. In cases such as these, Anwar’s identification as Muslim has been a subtextual element in the media reporting of the trials. More overtly, Anwar condemned Siddique’s 2007 conviction from the courthouse steps; for making that statement, he was charged with contempt of court. Anwar told me that Muslims were politically suspect at the time, just months after an attack on Glasgow Airport, and that because he and his client shared a religion, their institutional reception was similarly bundled. He says the judge identified his statement with his personal beliefs, not those of his client, and this inspired the contempt charge.

Finally, Anwar has made media relations a central part of his legal and campaigning work. After finishing his first degree, he worked briefly for BBC Scotland, where, he said, he was constrained from reporting on campaigns that he had organised. He decided to make the news in the colloquial rather than the denotative sense, hence his move into advocacy. The high profile of cases such as the Chhokar murder; the trial and appeal of Siddique; and the defamation lawsuit and subsequent perjury trial of politician Tommy Sheridan have brought him into frequent contact with journalists and, in his own words, made him ‘one of the most prominent lawyers with a media profile out there’. From 2012 to 2015, he wrote a weekly column for the Scottish edition of the *Sun*, which amplified his profile even as it complicated his relationships with Muslims and left-leaning activists in the city who disagree with the tabloid’s editorial stances.

In this constructed biography, we can begin to position Anwar within the network of relations between Muslims, civic institutions and the media in Glasgow. A field analysis of his media relations illuminates both internal and external dynamics. Anwar presented himself as an outlier: he spoke sneeringly of ‘community leaders’ among Glasgow’s Muslims who, according to him, appropriated a representative role without actually representing the views of the community. Similarly, he entered law after seeking redress for his violent treatment at the hands of authorities. As a ‘new entrant’ to these fields, he used subversion strategies to establish his position. He gained capital through his successes in student politics and in his court challenge, and by converting this capital through the study of law, he has transformed into an ‘authorised knower’ or, in Bourdieusien terms, an agent who has legitimacy. Though he sneered at Muslim ‘community leaders’ in our interview, he also acknowledged that he would by now be classed among them. Nevertheless, he has maintained the subversive elements of his habitus by defending clients of marginal status, championing causes that challenge the status quo among Glasgow Muslims and using his position to direct journalists to other active Muslims who do not enjoy the same degree of capital as he does. Anwar displays what Crossley (2003) calls the ‘radical habitus’, not merely reproducing but transforming both his positions in the relative fields and the fields themselves.

### Amina as New Entrant: Autonomy and Heteronomy

The staff and volunteers at AMINA – Muslim Women’s Resource Centre (hereafter Amina) are also agents working out source relations with journalists. Unlike Anwar, they were relatively new entrants during the course of my fieldwork. Amina provides social support services for women across Scotland who identify as Muslim. The centre has operated since 1997, but in terms of news coverage it maintained a low profile until the summer of 2012. During fieldwork, I observed members trying to capitalise on this sudden increase so as to fulfil their organisational goals.

If we again construct the media trajectory of the institution through successive states in its relation to other fields, its first taste of success followed a visit from Nicola Sturgeon, at the time the Scottish health secretary, in November 2011. She chose the centre as the venue to announce new legislation concerning forced marriage, and the centre’s director, Smina Akhtar, was quoted in many of the articles that reported the announcement. After this one-off event, news coverage of Amina returned to low levels until the following summer, when the centre generated even more coverage for a campaign called ‘I Speak for Myself’ that used compelling visual images to challenge perceptions about Muslim women. Before this campaign, members received media training from a former journalist employed to support groups in the Govanhill neighbourhood of Glasgow.

One effect of the training was an awareness among members of strategies: participants used journalistic language (e.g. stories having a ‘peg’ and getting ‘picked up’) during our interviews, analysed the news cycle and mentioned writing and disseminating press releases as a means of communicating with journalists. However, some of the members were ambivalent about the effectiveness of media relations and their ability to influence journalists. Social media were discussed as an alternative but unequal means to communicate. Direct and managed communications through Twitter, Facebook and blogs were useful for spreading news of their events or commenting on current affairs, generating ‘likes’, ‘clicks’ or ‘views’. However, Akhtar said such communications typically reached ‘the converted’, which she distinguished from ‘the public’. Her attempts to reach the latter relied on what she described at different times as the ‘traditional’ or ‘actual’ media. This terminology – especially the respect conferred by the word ‘actual’ – suggests that the mainstream media still mattered for Amina’s members despite their uneven attempts to control their relationship with these organisations and journalists. They did not possess the capital Anwar exhibited, and this can be explored in examples of attempts by each party to control the coverage of their activity.

### Media Relations in Practice

The day before my interview with Anwar, the lawyer had organised a press conference with his clients – the family of a Glasgow woman who had been murdered while visiting Pakistan with her husband. Anwar told reporters that police in Lahore had arrested a suspect and released his name to the Pakistani public. Journalists in Glasgow, however, were reluctant to publish the information: they feared that the release of the name was not verified, and their own lawyers urged caution before potentially interfering in an international investigation. Anwar said he was on the phone throughout the day, talking to journalists at both the private and public broadcasters and the print media. His intervention was direct:

[I was] giving him the number, saying, ‘[first name of broadcast editor], why don’t you phone the superintendent [in Lahore], get a recorded interview?’ Then when I’ve got confirmation one media’s recorded it, the editors of the other papers call me saying, ‘Aamer, we can’t run that. Because our lawyers are running scared.’ Then I call the lawyers and say, ‘Look, I’ve got that [covered]. You go back to them and say, “Look, they’re [the broadcasters] gonna run with it.” You need to run a piece.’ And very much the case that everybody had to run a piece and name him as a suspect, because I’ve got the [broadcasters] to do their reports.

Anwar’s account gives the impression of a stage manager, orchestrating all the media actors to contribute to the outcome he wanted – coverage that would help his clients.

Journalists had a professional obligation to cover significant steps in what was, despite its international elements, a local story. Journalists were also obliged, however, to adhere to the conventions of their trade, including the ‘strategic ritual’ of verification (Shapiro et al., 2013). Anwar said he had to be ‘to-ing and fro-ing and playing people off’ to make the name of the arrested suspect public. By co-ordinating the efforts of the various media in Glasgow, ‘everybody was able to run the story. But it was very much an operation of knowing what was going on where.’ Anwar had several levers at his disposal to influence the concerns of journalists. He was familiar with the timing of news routines and the different schedules of broadcast and print media; moreover, he knew the hierarchies that authorised stories, which positions to appeal to and what strategies to use to convince them. He demonstrated to me the intimacy of his relationships by referring to editors and media lawyers by their first names during our interview. Finally, he acknowledged that his position as a lawyer also helped – explicitly, by allaying the media lawyers’ concerns about identifying the suspect, and implicitly, in terms of the social capital his role carries.

Seen through Couldry’s lens, the institutions and agents which compose Glasgow’s journalistic field imposed their media meta-capital by influencing Anwar’s strategy for arguing his case in the legal field. In a general sense, the media orientation which he displayed throughout his legal and activist trajectory suggests media meta-capital at work. Anwar says he has had to learn to tone down his ideological commitments: ‘I’ve developed a pretty close working relationship with most of the media. I’ve also realised you can have your right wing, you can have your left wing, and I don’t put the phone down on any of them anymore.’ To manicure his media presence, he has also moderated his impulse to showy displays of outrage, ‘shouting and screaming as I come out of the courtroom. […] Learning to be controlled but very much packaged so I could get the point out to the maximum number of people.’ These changes show Anwar’s desire to present himself both to and through the media in an acceptable way.

Yet, as Bourdieu (2005) observed, the weak autonomy of the journalistic field makes it reliant on other agenda in decisions of coverage. In this example, journalists have learned the kind of stories Anwar offers and the tone of comment he is likely to give them. They know they can count on him to give a statement if they ask, and they know his political connections. If editors with one organisation had decided the publication of the suspect’s name was too risky, what would the implications have been when their competitors decided otherwise? What would their reticence mean the next time they needed a quotation or other assistance from Anwar, when they denied him coverage in this instance? Thus, the ability of journalists to impose their constraints on agents in other fields did not provide them with a corresponding autonomy. Anwar’s social capital – his position as an ‘authorised knower’ in legal matters and in relation to Muslims in Scotland – gave him considerable control in managing the publicity of his case. Being present in the media in this way allowed him to position himself among Scottish Muslims as a defender of their interests in a potentially uninterested Scottish civic sphere, thus increasing his social capital and shoring up his position in that field of relations.

Amina attempted to take similar control of the news agenda in promoting its work on violence against women, including the campaign ‘You Can Change This’. In April 2013, staff learned of an upcoming episode of BBC programme *Panorama* that investigated Sharia councils in England. Amina staff prepared a press release that responded to the programme, sharing information concerning the religious and civic rights Muslim women have in Scotland, material promoting their campaign, a video the centre had produced and quotations from staff and other Glasgow Muslims. Staff posted the release on the campaign’s blog and then publicised it through a series of tweets from Twitter account @ChangeThis_Scot. The account user had not tweeted in this way before, publishing a strategic release of several tweets targeting news media audiences, nor did she afterwards; this gave the series of tweets the look of an experiment, using a novel medium to deliver a conventional message.

Some of the tweets declared their purpose explicitly: two began with the phrase ‘PRESS RELEASE’, all in capital letters; a third began more informally with an invitation that included a hashtag – a tool for aggregating relevant terms on Twitter – saying, ‘Check out our latest #changemaker blog: Press Release’, followed by promotional material. Five other tweets were directed to specific Twitter accounts, beginning the message with the recipient’s handle. Some of these tweets courted individual journalists (e.g. @CatrinNye, @DaniGaravelli1), others general newsroom accounts (e.g. @scotsmannews, @ThirdForceNews). These tweets contained the identical message, ‘have you seen our press release on #BBCpanorama’s inside Sharia Councils?’ followed by a link.

Though it is possible for anyone to find any of these messages on the account of @ChangeThis_Scot, the first set of tweets would appear in the Twitter feed of all followers of the account, journalist or not. Beginning tweets with plain text is the most public way of tweeting, though the majuscule declaration ‘PRESS RELEASE’ might reduce its interest for those not working in the news media. The second set, beginning with a Twitter handle, would only appear in the feeds of Twitter users who follow accounts of both the campaign and the journalist or news organisation first mentioned in the tweet, reducing the public quality of the communication. In both cases, the recipient account holder would receive a notification that they had been mentioned in a tweet. These sets correspond to two typical strategies that participants in my study identified for proposing stories to journalists: press releases and targeted pitches. The former are sent by email to the accounts of newsrooms in the hope that an assignment editor will either use the material in a story or get in touch for more information. The latter are directed to specific journalists, and both sources and journalists told me that to be effective, the sender should identify journalists who have covered a particular kind of story or are correspondents with a relevant beat. The content of the pitch, while containing the same details as a press release, should be crafted to appeal to that journalist’s interests, including a personalised form of address, reminders where possible of prior communication between the sender and the journalist and appeals to the elements that made the story a ‘good fit’ for the journalist.

The first, widely disseminated set of tweets from @ChangeThis_Scot employed the first strategy. The second set of tweets employed both strategies, sometimes in the same tweet. Tweets in that set alerted specific journalists *and* the anonymous monitors of general accounts to the information Amina had to offer. I found no evidence of news stories that used Amina’s comment on Sharia courts, though I did find a Twitter exchange five months later between @ChangeThis_Scot and @DaniGaravelli1, the account connected to a journalist with daily broadsheet the *Scotsman*. This exchange, initiated by the user of Garavelli’s account, concerned identifying someone who could speak about wearing the niqab or face-covering veil in public institutions; the Twitter exchange ended with a decision to communicate via another medium (email), and several days later, Amina’s director was quoted in Garavelli’s story in the broadsheet’s Sunday equivalent, *Scotland on Sunday*. We cannot draw a straight line between the centre’s April tweets and the September quotation; the evidence that the one planted some seed that grew into the other is merely suggestive, lacking validity, but it could be interpreted as an eventual success of the experiment.

Scholarship on the uses of Twitter in journalism is young, but research is beginning to examine how it and other social media affect journalist–source relations. Peter Verweij (2012) examined Twitter relations and exchanges between politicians and journalists in the Netherlands. He found that ‘offline’ networks often underpinned social media interaction, that Twitter could not properly be understood in isolation from other technologies such as email or the telephone and that – in line with Bourdieu’s inter-field analysis – the parties were mutually reliant in accomplishing their respective work within their respective fields. Richard Waters and colleagues (2010), meanwhile, observed what they call ‘media catching’, in which public relations officials use social media to connect journalists with sources who can offer particular expertise.

However heteronomous the journalistic field is in relation to the political field, Amina’s Twitter exchanges constitute an instance in which journalists demonstrated their autonomy. Whereas *Panorama*’s national revelations about the advice Sharia courts gave British women were sensational and newsworthy, the contribution to local news production of a Scottish women’s centre seen primarily to serve a minority population was comparatively easy to ignore. The story, though, is not simply that the niche source is weak and the mainstream journalists are powerful: Anwar, as discussed above, has accrued significant social capital and has an authoritative relationship with journalists, though he comes from the same religious background as Amina. Amina itself has had successes – both active (e.g. the ‘I Speak for Myself’ campaign) and reactive (e.g. the Health Secretary’s visit in 2011). However, despite the presence of elements that are attractive to journalists, including a timely local angle on a national story, a clear message and targeted communication, the Twitter campaign did not generate coverage. Other attempts to promote the ‘You Can Change This’ campaign met with similar indifference: the campaign leader told me that her first attempts to market the video were ignored, even though it ‘had some pretty big names in there’ and novel features, responding to values she had learned during training. Amina promoted it with ‘a big press release. Done everything for them [the journalists], and no one picked it up. But, it may be there was something bigger that week, something else happened. So there’s nothing you can do about that.’ Weeks later, something big did happen: a rape and murder in India made violence against women a trending global story; the barrage of media phone calls to Amina about the campaign came over the Christmas holidays, when the centre was closed and staff were away.

Couldry’s modification of media meta-capital is pertinent here, suggesting the terms by which Amina members felt they had to articulate their messages in order to publicise them. Several staff and volunteers spoke of the need to be more energetic, to learn and fathom the requirements of journalists so as to improve their ability to respond effectively. They wanted to better appropriate the ‘internal rules and logics’ (Willig et al., 2015: 3) of the journalistic field. Akhtar, the director, was ambivalent, however: the energy her staff committed was not repaid in coverage, and she questioned how important coverage was for their goals. ‘Our main purpose is to do the work’, she told me, referring to Amina’s social support for Muslim women. ‘[I]f it [a press release] is taken up, fantastic. If it’s not, fine. It’s not really had an impact on the work. […] We don’t live for it [media coverage].’ With this statement, she attempted to demonstrate Amina’s autonomy as a group peripheral to the journalistic field. However, earlier in our interview, she articulated the benefits of being in the news, including recognition of her and her organisation’s name by funding agencies. The meta-capital which the news media exert on other fields presents both constraints and enables the work of actors in those fields.

## Conclusion

In this article, I have used data generated from fieldwork in Glasgow to illustrate the usefulness of Pierre Bourdieu’s field theory for understanding journalist–source relations. This is consonant with Bourdieu’s approach to both the media, which he viewed as heteronomous and intrinsically related to other fields, and to his theory generally, as interpreted by scholars such as Philip Schlesinger and Nick Couldry. Schlesinger uses Bourdieu’s emphasis on contingency, strategy and internal and external struggles to shake journalism studies from a media-centric approach to the field. Couldry acknowledges the pervasive quality of media and accounts for the influence of the journalistic field on supposedly discrete fields through the modification of media meta-capital. These tools offer us a way of thinking about collective activity in society, distinguishing the distribution of capital within a field and the relation between fields in what we might call wider society or, following Bourdieu, the field of power.

The rules by which fields are commonly understood can be too constraining for as diffuse a category as ‘sources’ or ‘Muslims’. Couldry, as well as Bottero and Crossley, have shown that a strict application of the concept can lead to contradictions or limit the useful application of the theory to empirical situations. By taking what Hilgers and Mangez (2014: 25) call ‘a pragmatic approach’, we can observe effects and relational dynamics in a field of strong social significance. To paraphrase Crossley (2003: 62), writing about protest and radical activism, ‘the world of [journalism] simply is that complex, and Bourdieu’s concept of fields […] is far better placed than any other tool in the sociological kit bag […] for engaging with and doing justice to this empirical complexity’.

The case of these two Muslim sources relating to journalists in Glasgow highlights that complexity. Aamer Anwar has retained his habitus as a transforming radical, developing this habitus through the nodes on his trajectory from student radical to campaigner to lawyer to Muslim ‘community leader’. The capital he has accrued in these fields gives him credit which he uses to communicate effectively with the media. A simple, unidirectional analysis, however, ignores his orientation towards the media which has pertained from the beginning of his public career, and it is his work in the media that has conferred much of the capital he now enjoys. Media meta-capital and Bourdieu’s tandem indices of autonomy and heteronomy help us understand his relations with journalists and media institutions.

Alternately, a series of tweets from Amina exposes challenges for a ‘new entrant’ in media relations. Media meta-capital constrains the members of Amina: they must negotiate their social support work in a media-saturated environment that is beyond their control. An experimental attempt to take hold of the news cycle and direct the attention of journalists their way failed, illustrating the relative autonomy of the journalistic field and the importance of social capital in exerting control on the media. This attempt also indicates the willingness of Amina to ‘play the game’ of journalism, though its effort was not rewarded.

Identifying and making sense of this complexity is important work for social research, especially concerning Muslims in Britain. The news media are implicated in the articulation and dissemination of anti-Muslim sentiment, but scholars who rely on content studies to derive conclusions about these representations miss exchanges that can enrich their analyses. Amina’s Tweets about the *Panorama* programme and Anwar’s ‘to-ing and fro-ing’ with editors about the naming of a murder suspect in Pakistan are invisible – a story that did not happen, strategic action that occurred outside the frame. To include them as data disrupts a facile conclusion that journalists systematically ignore or misrepresent Muslims in their coverage; we may find nuanced relationships or identify structures that need to change to improve those relations and, thereby, the content. The relative success of Amina and Anwar can in part be explained by considering their positions as new entrants and authorised knowers in a field of journalistic sources contributing to coverage of Muslims in Glasgow. An approach that examines journalist–source relations gives a denser portrayal of social interaction, challenging the construction of a binary opposition of journalists to Muslims that is an element of the contested concept of Islamophobia. The news media comprise one institution among many that negotiate and define the place of Muslims in British society. It is incumbent upon sociologists to give the media full and proper scrutiny, and Bourdieu’s writings provide tools to do so with rigour and creative attention.
